# 2195. Etiologies of medically attended acute respiratory infections among young Ecuadorian children prior to the start of the 2020 SARS-CoV-2 pandemic

**DOI:** 10.1093/ofid/ofac492.1814

**Published:** 2022-12-15

**Authors:** Manika Suryadevara, Freddy A Pizarro Fajardo, Cinthya Cueva Aponte, Jorge Carrillo Aponte, Cynthia Bonville, Irene Torres, Joseph B Domachowske

**Affiliations:** SUNY Upstate Medical University, Syracuse, New York; HOSPITAL TEOFILO DAVILA, Machala, El Oro, Ecuador; SUNY Upstate Medical University, Syracuse, New York; SUNY UPSTATE MEDICAL UNIVERSITY, QUITO, Pichincha, Ecuador; SUNY Upstate Medical University, Syracuse, New York; Fundacion Octaedro, Quito, Pichincha, Ecuador; SUNY Upstate Medical University, Syracuse, New York

## Abstract

**Background:**

Data regarding respiratory pathogen epidemiology in the tropical country of Ecuador are limited. Here, we describe the temporal patterns and etiologies of medically attended acute respiratory infections among Ecuadorean children during the 20-month period preceding the onset of the 2020 SARS-CoV-2 pandemic.

**Methods:**

Children < 5 years old presenting to a designated outpatient clinic with at least 2 new symptoms consistent with an acute respiratory infection are eligible for enrollment. Informed consent is obtained. Demographic and clinical details are collected. A nasopharyngeal sample is collected for diagnostic testing of 22 target pathogen groups using Biofire’s Respiratory Panel v1.7 multiplex polymerase chain reaction assay.

**Results:**

Of the 820 subjects enrolled between July 15, 2018 and March 15, 2020, 655 (80%) tested positive for at least one pathogen. The detection of pathogens was more likely from samples collected from children enrolled in Quito (85%) compared to Machala (76%) (p < 0.05). The most frequently detected pathogen groups were rhinovirus/enterovirus (46%), parainfluenza virus (14%), respiratory syncytial virus (RSV) (12%), and influenza virus (10%). Two or more pathogen groups were co-detected in 174 (27%) of the respiratory samples. Pathogen specific seasonal patterns were not observed for rhinovirus/enterovirus, adenovirus, or atypical bacteria at either site. Samples collected in Quito were positive for the detection of RSV spanning a 32-week period between November and June. In contrast, detection of RSV from samples collected in Machala spanned only a 17- week period between February and May. In Quito, influenza viruses were detected between August and February, with influenza A activity preceding that of influenza B. In Machala, the detection of influenza B virus coincided with the dry season, while detection of influenza A virus was clustered in the rainy period between January and March.

**Conclusion:**

The specific etiologies and seasonality of acute respiratory tract infections among Ecuadorean children < 5 years of age differ by site of enrollment. Such differences in regional data can be used to optimize regional implementation of existing and soon-to-be available public health prevention measures.

**Disclosures:**

**Manika Suryadevara, MD**, Janssen: Grant/Research Support|Merck: Grant/Research Support|Roche: Grant/Research Support.

